# Expanding Access to Retinal Imaging Through Patient-Operated Optical Coherence Tomography in a Veterans Affairs Retina Clinic

**DOI:** 10.3390/bioengineering13010061

**Published:** 2026-01-05

**Authors:** Alan B. Dogan, Katherine G. Barber, Brigid C. Devine, Blanche Kuo, Colin K. Drummond, Ankur A. Mehra, Eric S. Eleff, Warren M. Sobol

**Affiliations:** 1 Virginia Tech Carilion School of Medicine, Roanoke, VA 24016, USA; 2 Louis Stokes Cleveland Department of Veterans Affairs Medical Center, Cleveland, OH 44106, USA; katherine.barber@va.gov (K.G.B.); eric.eleff@va.gov (E.S.E.);; 3 College of Medicine, Northeast Ohio Medical University, Rootstown, OH 44272, USA; bdevine2@neomed.edu; 4 Department of Ophthalmology and Visual Sciences, Case Western Reserve University School of Medicine/University Hospitals Cleveland Medical Center, Cleveland, OH 44106, USA; 5 Department of Biomedical Engineering, Case School of Engineering School of Medicine, Cleveland, OH 44106, USA; drummond@case.edu

**Keywords:** optical coherence tomography, retinal imaging, Telehealth, screening, self-operated imaging, referral triage, community eye care

## Abstract

This study evaluated the feasibility, image quality, and referral accuracy of a patient-operated optical coherence tomography (OCT) device (SightSync) compared with technician-acquired Heidelberg OCT. This study was conducted in a Veterans Affairs retina clinic (Cleveland, Ohio), resulting in a predominantly male (98%) study population representative of the local veteran demographics. One hundred patients attempted self-administered OCT imaging after brief instruction, yielding 118 successful scans (59% of eyes) with no significant association between scan success and age, visual acuity, or diagnosis. Quantitative analysis of 142 paired images showed that SightSync produced interpretable scans with comparable sharpness to Heidelberg OCT, though signal- and intensity-based metrics (signal-to-noise ratio; SNR, contrast-to-noise ratio; CNR, entropy, pixel intensity; p90) were lower, consistent with hardware differences between a compact patient-operated prototype and a clinical-grade system. Among 121 high-quality SightSync scans, referral decisions demonstrated strong agreement with Heidelberg OCT, with a sensitivity of 83.9%, specificity of 75.6%, and a negative predictive value of 93.2%, indicating reliable exclusion of clinically significant pathology. These findings demonstrate that patients can independently acquire clinically interpretable OCT images and that SightSync provides safe, conservative triage performance—supporting its potential as a scalable community-based retinal imaging solution—while a review of unsuccessful scans has identified prototype modifications expected to further improve device feasibility.

## 1. Introduction

Optical coherence tomography (OCT) is central to modern retinal care, providing noninvasive, high-resolution imaging that guides diagnosis and treatment in conditions such as neovascular age-related macular degeneration (nAMD) and diabetic retinopathy (DR). Therapeutic decisions, such as whether to administer an anti-VEGF injection or defer treatment, often hinge on OCT findings like the presence of intraretinal or subretinal fluid. Despite the transformative role of OCT, access to imaging remains uneven, and adherence to regular follow-up is challenged by logistical and socioeconomic barriers [1,2]. Retina clinics are increasingly strained by the growing prevalence of chronic retinal disease, with visit volumes rising faster than the available retina workforce [3,4]. Missed or delayed imaging visits are a major driver of irreversible vision loss in nAMD and DR, highlighting the need for more accessible ways to obtain OCT data [5,6].

The burden of retinal disease is substantial. In the United States, age-related macular degeneration (AMD) and diabetic retinopathy (DR) together account for the majority of vision-threatening retinal disease. AMD affects ~6.5% of adults over 40, with nearly 1% having late-stage disease, and prevalence rises steeply with age [7]. DR is one of the most common complications of diabetes, affecting more than one-third of patients worldwide and causing diabetic macular edema in about one in ten [8,9,10]. Despite screening recommendations, nearly one in four people with diabetes do not obtain annual eye exams [11,12]. Asymptomatic progression, combined with logistical barriers, means that many cases remain undiagnosed until advanced, irreversible stages. Access to OCT imaging is particularly limited for rural residents, older adults, individuals with mobility or transportation challenges, and patients receiving care in the VA system, underscoring that the current clinic-centered model is insufficient to meet the monitoring needs of the growing at-risk population [13,14,15,16].

Adherence to routine retinal care is further undermined by real-world challenges. Patients frequently cite transportation, scheduling, and time away from work as barriers to attending appointments [17]. Poor follow-up is consistently linked to worse visual outcomes in retinal disease [2]. In real-world anti-VEGF cohorts, up to 22% of patients with neovascular AMD and 25% of those with DR experience lapses in follow-up lasting a year or more; these disruptions are not only common but directly detrimental, leading to irreversible vision loss in otherwise treatable disease [18,19,20].

Because OCT findings often drive medical and surgical management, expanding access to imaging has become a critical focus. Recent home OCT systems, such as the Notal Vision Home OCT, have demonstrated that patients can successfully self-image and that remote data can be used to guide fluid management in nAMD [21,22,23,24]. However, widespread deployment of home OCT is limited by cost, single-user assignment, distribution, and maintenance requirements, making it difficult to scale to the millions who require ongoing monitoring [25,26].

A scalable, low-cost OCT solution capable of functioning without technician support could meaningfully reduce barriers to routine monitoring. To address these challenges, we developed SightSync, a patient-operated OCT platform intended for community deployment rather than single-user home use [27]. By situating devices in accessible locations such as pharmacies, nursing homes, or Veterans Affairs Community-Based Outpatient Clinics (CBOCs), SightSync reduces per-patient cost and enables shared access without the need for a technician, with each unit priced at approximately $9000 [27,28]. Patients can acquire scans independently with minimal instruction, and images are securely transferred for physician review. This community-based model is designed to provide a scalable, cost-effective solution for monitoring—and ultimately screening—retinal pathology.

In this study, we evaluated the feasibility of patient-operated OCT, SightSync, in a retina clinic environment. Specifically, we tested whether patients could successfully obtain scans with only minimal instruction, and whether these scans—when compared against gold-standard technician OCT—were of sufficient quality to appropriately capture retinal pathology. We examined the overall success rate of patient-acquired scans, identified demographic and clinical factors influencing successful imaging, and compared image quality and diagnostic performance to standard clinical imaging. Ultimately, this pilot trial evaluates the viability of patient-operated OCT, identifies the key limitations and common failure modes of self-imaging, and provides critical insights to guide future refinements and broader deployment of this technology.

## 2. Materials and Methods

This study was approved by the Institutional Review Board of the Louis Stokes Cleveland Veterans Affairs Medical Center (IRB #1839081-3; “SightSync: Self-Monitoring Optical Coherence Tomography Device for Ophthalmology Retinal Telehealth”). All procedures adhered to the tenets of the Declaration of Helsinki and relevant Veterans Health Administration research policies. Written informed consent was obtained from all participants prior to enrollment.

Patients were recruited during their scheduled retinal eye exam at the Louis Stokes Cleveland VA Medical Center Eye Clinic (at the VA Northeast Ohio Healthcare System) using the inclusion/exclusion criteria (Figure S1). During the patient’s consultation, the VANEOHS ophthalmologist asked if the patient would be interested in participating in the research study. The ophthalmologist or the research coordinator described the study and obtained written consent in a private area. Following the scans, the patient completed a questionnaire regarding the comfort and usability of the SightSync device. Then, the VANEOHS ophthalmologist performed the scheduled comprehensive eye exam, including the standard OCT scan using the Heidelberg OCT device obtained by a trained VA technician.

### 2.1. SightSync OCT

The patient received an in-depth explanation of how to operate the machine prior to use, including step-by-step instructions. The patient logged into the SightSync device and obtained the OCT imaging of each eye using the self-scan OCT machine. Limited patient instructions were given for troubleshooting after initial instructions were provided. Patients were given up to 4 attempts to obtain a good scan, however the patient was able to stop the trial whenever they desired (e.g., if frustrated, time-constrained, etc.). On average, patients required 2.3 attempts to obtain a scan, though up to five attempts were permitted depending on patient time and willingness. The OCT scan was saved and securely transferred to the VA network into a HIPAA compliant shared cloud drive.

Instructions provided to the participants are shown in Figure S2.

### 2.2. Heidelberg OCT

Optical coherence tomography (OCT) imaging was performed using a Spectralis OCT system (Heidelberg Engineering GmbH, Heidelberg, Germany; Spec-CAM-15376-S3610). All scans were acquired by trained ophthalmic technicians following standard operating procedures for retinal imaging.

Technicians obtained macula-centered raster scans in accordance with manufacturer-recommended acquisition protocols. Each scan series consisted of high-resolution, averaged B-scans optimized for foveal centration and image quality. The imaging parameters, including scan angle, averaging, and focus adjustment, were standardized across subjects to ensure consistency in resolution and contrast.

Following acquisition, raster images were hand-selected by the study team to focus on frames centered around the macula. Images exhibiting motion artifact, poor signal strength, or inadequate fixation were excluded. Only high-quality scans meeting predefined signal-to-noise and centration criteria were included for downstream analysis.

Each image acquisition environment (SightSync vs. Heidelberg) is shown in Figure S3.

### 2.3. Data Collection Protocol and Image Rating

Images were deidentified and reviewed by three independent reviewers (Authors AD, BD, WS) for quality (“good” vs. “bad”). Images were programmatically organized into 2 × 2 collages including both SightSync and Heidelberg images (Figure S4).

Two blinded reviewers (Authors AAM and BK) conducted an independent review to assess the visibility pathologic findings in both the Heidelberg and Sightsync images determining whether or not the respective scans had sufficient information to (1) make a referral decision and (2) what that referral decision (should be seen vs. should not be seen) is based on the scan and other patient demographics. Indeterminate classifications were treated conservatively as referral-positive.

### 2.4. Statistical Analysis

A *p*-value of <0.05 was considered statistically significant unless otherwise specified. All analyses were conducted in Python 3.12.8 using scipy (v1.15.2), scikit-learn (v1.5.1), and statsmodels (v0.14.4) for statistical testing and model development. Data processing and visualization were performed using pandas (v2.2.3), numpy (v2.0.0), matplotlib (v3.10.0), and seaborn (v0.13.2). Diagnostic agreement metrics (sensitivity, specificity, PPV, NPV) were calculated directly from confusion matrix outputs and uncertainty was quantified using Wilson score 95% confidence intervals. All plotting functions were generated programmatically, and all code used for analysis is reproducible with the imported packages. The complete software environment, including package versions and configuration files, is available from the authors upon request.

## 3. Results

### 3.1. Experimental Setup and Enrollment

**Figure 1:**
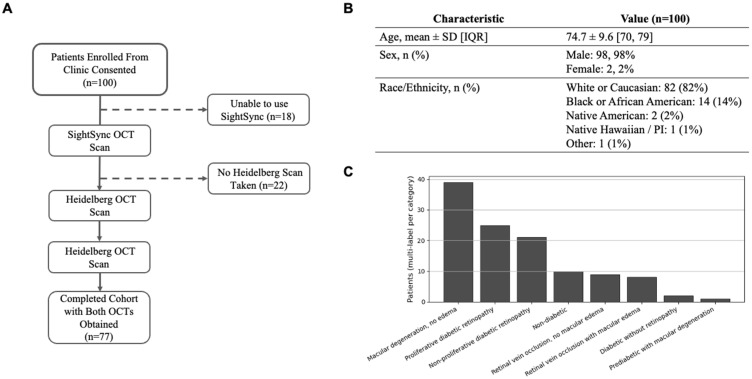
Study cohort characteristics and distribution of retinal diagnoses. (**A**) Participant flow from initial enrollment to the final cohort completing both SightSync and Heidelberg OCT scans. Eighteen patients were unable to obtain a SightSync scan and twenty-two did not undergo a Heidelberg scan, resulting in a final analytic cohort of 77 patients with paired OCT imaging. (**B**) Participant characteristics including age, sex, and race/ethnicity (**C**) Patient-level diagnosis categories. 35 patients within the cohort had >2 main retinal diagnoses.

We consecutively recruited 100 patients from the ophthalmology waiting room at the Louis Stokes VA. Of these, 18 participants were unable to complete an attempt at taking a SightSync scan, most commonly due to clinic workflow constraints (e.g., unwillingness to wait or being recalled by the provider for ongoing care) or difficulty achieving positioning related to mobility or cognitive limitations. Reference Heidelberg OCT imaging was acquired in 78 patients (78%); 22 patients (22%) did not undergo Heidelberg imaging because it was not clinically indicated for their visit. The overlap yielded 77 patients (77%) with paired imaging on both systems, which constituted the analytic cohort (Figure 1A). Patient demographics including age, sex and race/ethnicity is summarized in Figure 1B.

Clinical labels were assigned on a multi-label basis (patients could fall into more than one category). The most common diagnoses among the recruited patients were: macular degeneration without macular edema (n = 49), proliferative diabetic retinopathy (n = 30), diabetic without retinopathy (n = 23), non-diabetic (n = 12), retinal vein occlusion with macular edema (n = 12), non-proliferative diabetic retinopathy (n = 11), retinal vein occlusion without macular edema (n = 10), prediabetic with macular degeneration (n = 9), other retinal pathology (n = 2), and other (n = 1) (Figure 1C).

### 3.2. Scan Success Rate Segmented by Age, Visual Acuity and Primary Diagnosis

Across the 82 participants (164 eyes) who successfully obtained a SightSync, 118 eyes (72%) were successfully scanned using the patient-operated SightSync OCT device. At the participant level, 81.7% obtained a usable scan in ≥1 eye, and 51.2% achieved success in both eyes.

**Figure 2:**
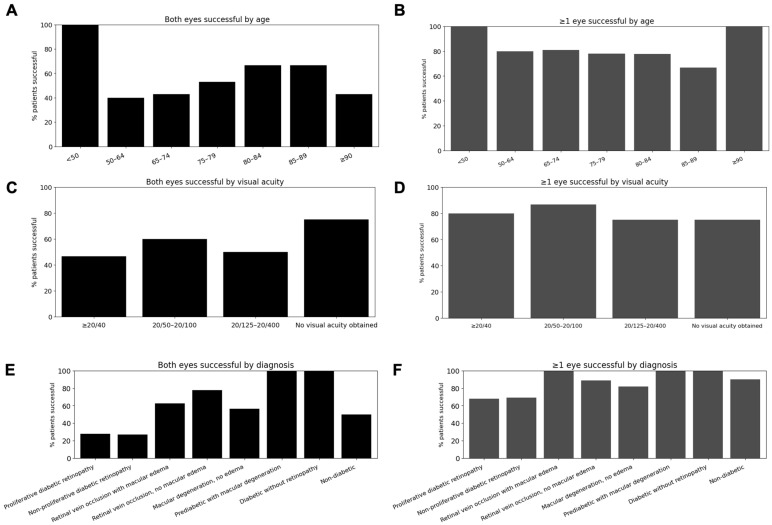
SightSync scanning success by demographic and clinical factors. (**A**,**C**,**E**) depict the percentage of participants who successfully completed scans in both eyes, while (**B**,**D**,**F**) show success in at least one eye. Scanning success was stratified by (**A**,**B**) age group, (**C**,**D**) visual acuity, and (**E**,**F**) primary diagnosis category. Across variables, there were no statistically significant associations with success: age (χ^2^, *p* = 0.755; linear trend *p* = 0.623), visual acuity (χ^2^, *p* = 0.603; linear trend *p* = 0.242), or diagnosis (all FDR-adjusted *p* > 0.29).

By age, ≥1-eye success ranged from 66.7% (85–89 years: 2/3) to 100% (<50 years: 1/1; ≥90 years: 7/7), with most age groups demonstrating success rates between ~75–81% (e.g., 50–64: 8/10; 65–74: 17/21; 75–79: 25/32; 80–84: 7/9). There was no significant association between age and either bilateral success (χ^2^ *p* = 0.755; linear trend *p* = 0.623) or ≥1-eye success (χ^2^ *p* = 0.870; linear trend *p* = 0.615). These results are shown in Figure 2.

By visual acuity, ≥1-eye success was similarly consistent across strata—80.0% (≥20/40: 48/60), 86.7% (20/50–20/100: 13/15), 75.0% (20/125–20/400: 3/4), and 75.0% (no visual acuity obtained: 3/4)—with no significant differences for either bilateral success (χ^2^ *p* = 0.603; trend *p* = 0.242) or ≥1-eye success (χ^2^ *p* = 0.913; trend *p* = 0.889).

When stratified by diagnosis, ≥1-eye success did not differ significantly across categories (all FDR-adjusted *p* ≥ 0.29). However, bilateral scanning success was significantly associated with diagnosis, with lower odds observed in proliferative and non-proliferative diabetic retinopathy compared with other diagnoses (both FDR-adjusted *p* = 0.027). No other diagnostic categories demonstrated significant associations after correction for multiple comparisons.

### 3.3. Questionnaire Feedback Survey

Out of the 100 participants, 98 individuals completed a five-item questionnaire feedback survey which assessed the quality of user experience during patient-operated scanning with the SightSync OCT device. Two of the questions prompted categorical responses of “Yes,” “Somewhat,” and “No,” one question prompted categorical responses of “Yes” and “No,” and two questions used 5-point Likert scales to assess comfort using the device (from “Very uncomfortable” to “Very comfortable”) and satisfaction of overall experience with the device (from “Very dissatisfied” to “Very satisfied”). These survey results were analyzed for differences between patients who had successful versus unsuccessful image acquisitions using the SightSync OCT device.

**Figure 3:**
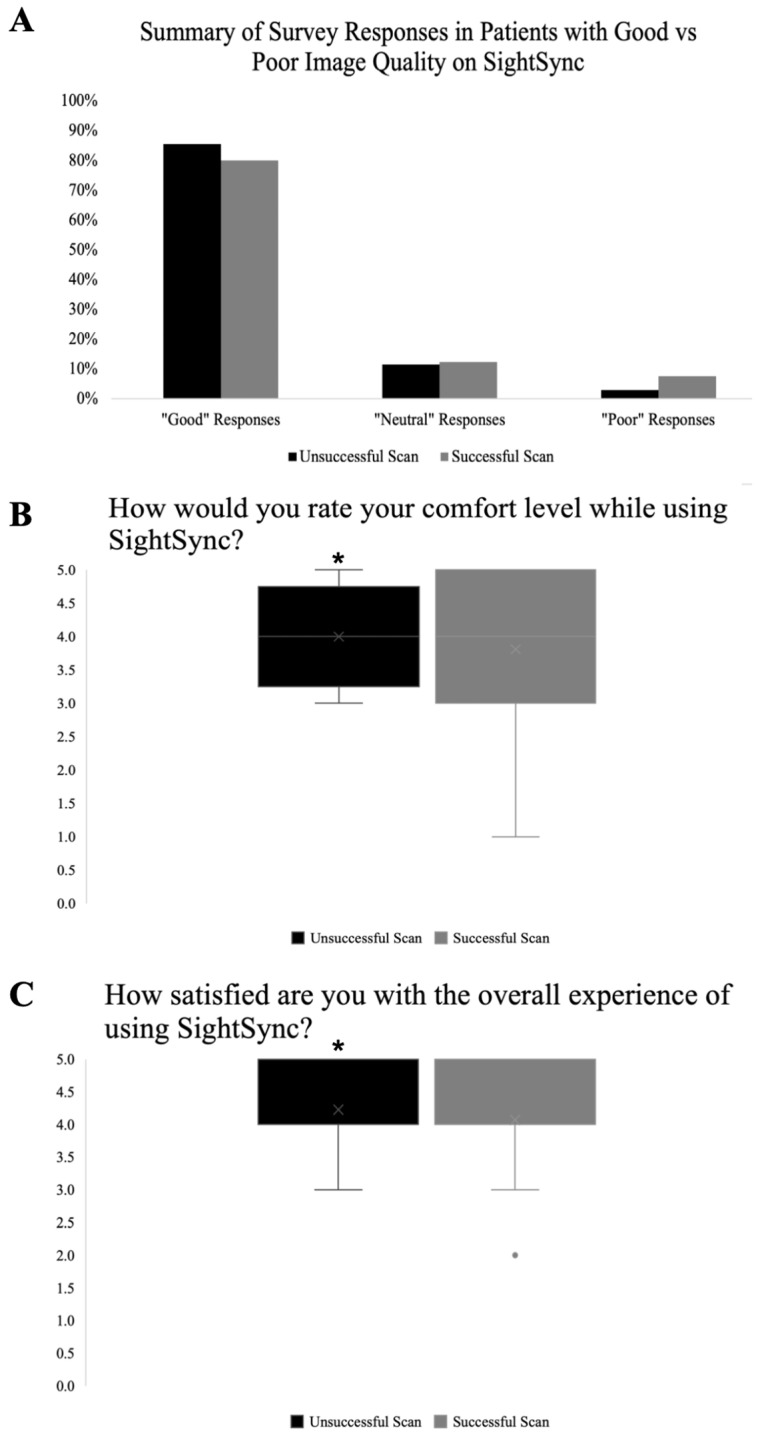
Comparison of questionnaire feedback survey data of participants with unsuccessful scans versus successful scans. (**A**) Summative data comparing “good,” “neutral,” and “poor” responses from all five survey questions shows no association of overall response data success of scans. (**B**) Box-and-whisker plot showing participant rating of comfort level while using the SightSync OCT device and (**C**) satisfaction with overall experience of using the SightSync OCT device. Patients with successful scans had significantly higher ratings of comfort (Mann–Whitney U = 240, *p* < 0.001) and satisfaction (Mann–Whitney U = 358, *p* < 0.001). Boxes represent the interquartile range (IQR), horizontal lines denote medians, and whiskers show data range excluding outliers. * *p* < 0.001 via Mann–Whitney U.

Overall response data, classified into “good,” “neutral,” and “poor” responses were stratified between patients with successful and unsuccessful scans for analysis and are represented in Figure 3A.

Among categorical data, the distribution of responses was similar between participants who had successful scans and participants who had unsuccessful scans. A chi-square test of independence showed no significant association between scan success and impression of the device (comparing “good,” “neutral,” and “poor” responses of categorical data), X2 (2, N = 294) = 1.71, *p* = 0.42.

For Likert-scale questions, Mann–Whitney U tests were used to compare comfort and satisfaction scores between groups (Figure 3B,C). Comfortability ratings were significantly higher among participants with successful scans (median = 4, IQR = 3–4.75) compared to participants with unsuccessful scans (median = 4, IQR = 3.75–4.25), U = 240, *p* = 2.93 × 10^−11^. Similarly, satisfaction ratings were significantly higher among participants with successful scans (median = 4, IQR = 4–5) compared with participants with unsuccessful scans (median = 4, IQR = 4–5), U = 358, *p* = 6.75 × 10^−9^. Cliff’s delta was then calculated to further assess the effect size of these differences in response data between patients with successful and unsuccessful scans and was found to be 0.384 for comfortability and 0.366 for satisfaction. This indicates there is a moderate effect, suggesting that both comfort and satisfaction ratings are meaningfully higher among patients with successful scans.

### 3.4. Quantitative Image Quality Comparison Between SightSync and Heidelberg OCT

Image quality is paramount for meaningful pathological analysis in OCT; poor signal, contrast, or sharpness can obscure clinically relevant findings and degrade diagnostic confidence. We therefore tested whether ‘successful’, patient-obtained SightSync scans deliver image quality comparable to technician-acquired clinical OCT by directly comparing core metrics (SNR, CNR, sharpness, entropy, and p90).

A total of 142 paired scans from 100 participants were analyzed to compare image quality metrics between the SightSync prototype and the clinical Heidelberg Spectralis OCT system. Metrics included linear signal-to-noise ratio (SNR), contrast-to-noise ratio (CNR), Laplacian variance (sharpness), entropy, and the upper-tail intensity percentile p90 (the lower-tail percentile p10 was not analyzed due to a floor effect with values clustering near zero). Outliers exceeding three standard deviations were excluded before analysis.

**Figure 4:**
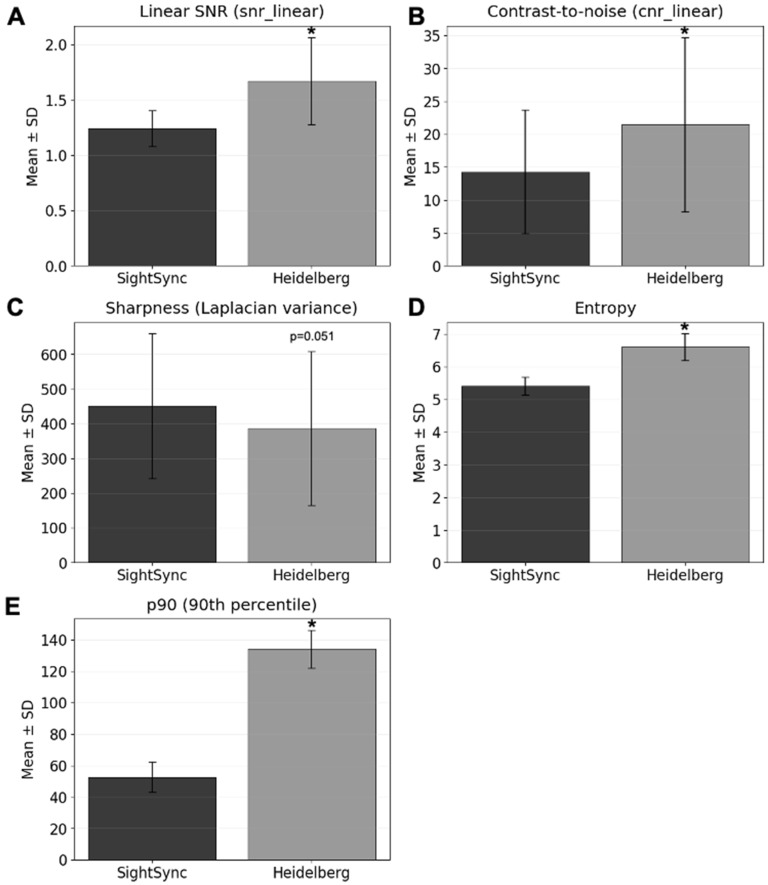
Comparison of image quality metrics between the SightSync and Heidelberg OCT systems. (**A**) Linear signal-to-noise ratio (SNR) and (**B**) contrast-to-noise ratio (CNR) reflecting signal intensity and contrast resolution within retinal layers. (**C**) Sharpness, quantified as Laplacian variance, indicating comparable edge clarity and focus. (**D**) Entropy, a measure of image texture complexity and (**E**) the 90th-percentile intensity (p90) reflects the upper tail, representing the brightest signal regions such as highly reflective retinal interfaces. Error bars denote standard deviation. * denotes *p* < 0.01 via paired *t*-test.

The Heidelberg OCT demonstrated significantly higher linear SNR (mean ± SD: 1.674 ± 0.394 vs. 1.240 ± 0.162, t(100) ≈ 12.0, *p* = 1.024 × 10^−17^, 95% CI [0.36, 0.50]) and higher CNR (21.50 ± 13.25 vs. 14.31 ± 9.40, *p* = 3.495 × 10^−4^, 95% CI [3.3, 11.1]) compared with SightSync (Figure 4A,B). No significant difference was observed in sharpness (Laplacian variance; 386.6 ± 221.3 vs. 450.8 ± 208.1, *p* = 0.05071, 95% CI [–128.6, 0.2]) (Figure 4C). Entropy was significantly higher for Heidelberg (6.608 ± 0.408 vs. 5.415 ± 0.278, *p* = 1.002 × 10^−46^, 95% CI [1.00, 1.39]) (Figure 4D). For intensity percentiles, we report p90—corresponding to the brightest retinal interfaces—which was markedly higher on Heidelberg (134.0 ± 11.91 vs. 52.64 ± 9.76, *p* = 2.68 × 10^−76^, 95% CI [68.07, 94.65]) (Figure 4E); p10 was omitted from primary analyses due to a pronounced floor effect and limited discriminative value.

### 3.5. Agreement Between SightSync and Heidelberg in Referral Assessment

To evaluate the ability of the SightSync device to identify patients requiring ophthalmic follow-up, referral classifications were compared directly with those from Heidelberg OCT using two blinded graders. Classification performance metrics (sensitivity, specificity, PPV, and NPV) were calculated conditional on successful image acquisition. Using only high-quality SightSync scans (N = 121 eyes) and after excluding indeterminate Heidelberg scans, sensitivity ranged from 80.5% to 83.9%, while specificity ranged from 75.6% to 90.0%, reflecting consistent diagnostic performance across graders with differing referral thresholds. Inter-grader agreement for Heidelberg-based referral classification was substantial (Cohen’s κ = 0.80), with disagreement observed in 10 of 121 cases (8.3%), supporting the reliability of the reference standard.

When evaluated against a conservative consensus reference—defined as referral-positive if either grader indicated referral—SightSync achieved a sensitivity of 70.7% (95% CI, 55.5–82.4%) and a specificity of 76.2% (95% CI, 65.9–84.2%). Positive predictive value was 60.4% (95% CI, 46.3–73.0%), while negative predictive value remained high at 83.6% (95% CI, 73.4–90.3%). Overall accuracy under the consensus framework was 74.4%.

However, because SightSync achieved a successful acquisition rate of 72%, end-to-end (“effective”) screening performance was calculated under the conservative assumption that uninterpretable scans would prompt referral. Under this framework, effective sensitivity and specificity were 50.9% and 54.9%, respectively, with an effective positive predictive value of 43.5% and a negative predictive value of 83.6%.

## 4. Discussion

The enrollment cohort (Figure 1A) reflects a typical veteran retina clinic population and frames interpretation of the feasibility results. Demographically, participants showed broad representation across the decades most affected by retinal disease—with substantial numbers in their 60s, 70s, 80s, and ≥90—providing an age distribution well aligned to real-world risk (Figure 1B). Diagnostically, multi-label assignment revealed that many patients carried more than one retinal pathology (35 with ≥2 primary categories), highlighting the complexity of routine presentations and the need for imaging pathways that perform consistently amid mixed disease (Figure 1C). A key limitation is sex balance: recruiting exclusively at a Veterans Affairs facility yielded a predominantly male sample (Figure 1B), which may limit generalizability to settings with more balanced sex representation. Even so, the breadth of ages and the high prevalence of overlapping pathologies observed here provide a realistic foundation for evaluating patient-operated OCT in subsequent analyses.

In the scanned cohort of 82 patients, 118 eyes (72%) were successfully scanned using the SightSync OCT device. Commonly cited device failures involved (1) inability to fixate on the internal red fixation target; (2) incorrect focal positioning, in which users were positioned too close to or too far from the sensor, causing the retinal signal to fall outside the optimal focal and zero-delay range—resulting in blank images or axial image inversion due to Fourier-domain reconstruction ambiguity; and (3) incomplete understanding of device instructions. Future iterations incorporating a larger, higher-salience fixation target, enhanced video-based user guidance, and a dynamic focal-length sweep are expected to substantially reduce these scanning failures. No significant associations were observed between scanning success and age or visual acuity. The scan success rate observed in this study is lower than that reported for Notal Vision’s Scanly Home OCT (96% success across 160 subjects in a trained home-use cohort); however, that system relies on a static, table-top design and established training workflows that may not generalize to lower-cost, community-deployed settings [22]. Notably, more than one-third of enrolled patients had multiple (≥2) concurrent retinal pathologies, which likely contributed to the lower observed success rate by reflecting a study population enriched for extensive retinal disease and associated comorbidities.

Subgroup analyses across age, visual acuity, and diagnostic categories were conducted to explore potential trends in scan success rather than to establish definitive predictors of performance. These analyses were not powered for formal inference, and despite correction for multiple comparisons, residual risks of false-positive and false-negative findings remain. The absence of significant associations should therefore be interpreted cautiously, particularly given modest sample sizes within individual strata and the exploratory nature of these comparisons. Age-stratified results (Figure 2A,B) showed stable success across decades, with even the ≥90-year group achieving 100% success, suggesting that age itself is not a limiting factor. However, cognitive ability—unmeasured in this study —may be an underlying determinant of scan success among older participants. Visual acuity (Figure 2C,D) had minimal impact, as individuals with ≥20/40 vision performed similarly to those with 20/125–20/400, indicating that moderate visual impairment does not preclude accurate self-alignment despite a small fixation target. Diagnostic segmentation (Figure 2E,F) revealed lower bilateral scanning success in proliferative and non-proliferative diabetic retinopathy, reaching statistical significance despite limited power, while ≥1-eye success remained high across diagnoses.

Overall, 82.2% of survey results reported positive feedback, indicating that most patients had good experiences using the SightSync (Figure 3A). There was not a significant difference in categorical survey response results between patients who had a successful versus an unsuccessful scan (Figure 3A, *p* < 0.42). However, there was a statistically significant difference in the Likert-scale responses, indicating that there is a meaningful shift toward higher comfort and satisfaction ratings in patients with successful scans. Categorical questions collected specific data regarding the user-perceived invasiveness of the device, whether the instructions were easy to follow, and whether the user experienced any issues during the scan. Since these response data did not vary with scan success, it is possible that the scan success rate may be attributed to other factors, such as cognitive impairment, in which the patient may be unaware of scanning difficulties or other measures of experience. Additionally, more subjective questions showed that higher comfort and satisfaction was associated with successful scans; these perceptions of overall experience may be more likely influenced by whether the user was able to attain a successful scan output on the first effort. Further study of the association between objective measures of cognition (such as the Mini-Mental State Exam or Montreal Cognitive Assessment) may be used to better understand the relationship between cognitive decline and scan success. Especially since the population receiving these scans is largely made up of elderly patients, this knowledge could help to identify patients most likely to benefit from the SightSync.

Quantitative image analysis demonstrated that the patient-operated SightSync OCT is capable of producing clinically interpretable images, though technician-acquired Heidelberg OCT scans achieved higher overall signal quality. The SightSync system exhibited lower mean SNR and CNR values (Figure 4A,B), indicating comparatively reduced signal uniformity and contrast across retinal layers. This difference likely reflects hardware disparities between a compact, patient-operated prototype and the high-sensitivity photodetectors and averaging capabilities of a clinical-grade system. Despite this, no significant difference in image sharpness (Figure 4C) was observed, suggesting that focus and optical alignment were adequately maintained during self-administered scans.

Entropy and high-intensity percentile (p90) values (Figure 4D,E) were significantly higher in Heidelberg images, consistent with the system’s superior dynamic range and illumination stability. These findings collectively highlight the inherent trade-off between accessibility and signal performance—while clinical systems deliver optimal quantitative image quality, SightSync can achieve usable results in an unassisted setting.

The modest performance gap in intensity-based metrics suggests that future hardware iterations may benefit from optimized light source power, detector gain tuning, and adaptive exposure control. Moreover, algorithmic denoising or image averaging could further narrow the difference in signal quality without compromising the device’s portability or ease of use. Overall, these results support the feasibility of community-deployed, patient-operated OCT imaging while identifying key engineering pathways to approach clinical-grade image quality in future SightSync versions.

**Figure 5:**
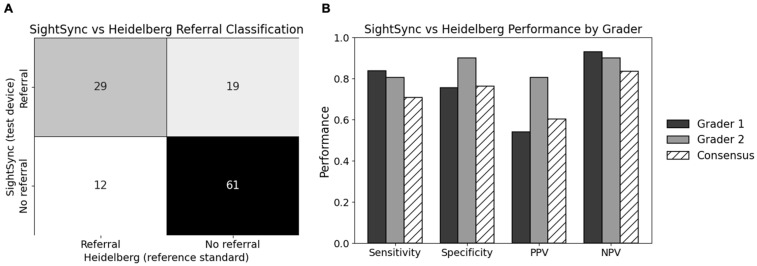
Classification agreement between SightSync and Heidelberg OCT for determining whether a patient should be referred for ophthalmic evaluation. (**A**) Confusion matrix depicting concordance and discordance between SightSync referral classifications and the Heidelberg reference standard. (**B**) Bar plot summarizing key diagnostic performance metrics, including sensitivity, specificity, positive predictive value (PPV), and negative predictive value (NPV).

The referral-classification results highlight the feasibility of how a patient-operated OCT can support safe triage in decentralized care settings, assuming the frequency of high-quality image acquisition is increased in future iterations of the device. The confusion matrix (Figure 5A) shows a performance pattern well aligned with a screening-first device; SightSync achieved strong sensitivity and a high negative predictive value, indicating that when the system obtains a sufficiently high-quality scan it can reliably identifies eyes unlikely to require in-person evaluation. However, this analysis included only high-quality scans as defined by the selection process in Figure 2. A truly deployable patient-operated OCT system will require higher overall scan success rates or real-time user feedback to guide alignment and improve image acquisition. Accounting for acquisition failures reduced effective PPV, sensitivity and specificity due to increased referrals but did not materially affect NPV, as uninterpretable scans were conservatively classified as screen-positive and therefore did not contribute to false negatives. Accordingly, future studies will focus on improving usability and evaluating whether optimized system iterations can achieve reliable pathology detection and patient triage.

The relatively low effective positive predictive value and presence of false-positive alerts (Figure 5B) are expected in a conservative triage workflow; however, improvement in successful scan frequency will be paramount to eventual deployment of the device. These overcalls likely stem from user-driven alignment variability and subtle image artifacts inherent to self-administered scanning, and it is anticipated that enhanced autofocusing and advanced image-processing techniques (e.g., super-resolution, histogram matching) will ultimately improve image quality and increase the positive predictive value. However, this shift toward increased referrals underscores a key limitation of the current system: false-positive alerts impose additional clinical burden and must be substantially reduced before widespread deployment, as unnecessary confirmatory examinations could strain clinical workflows and undermine scalability.

These findings suggest clear avenues for refinement—including improved user feedback, real-time quality scoring, and automated structural analysis—to further reduce false positives. Overall, SightSync demonstrates the feasibility of a safe and effective referral-screening profile, supporting its potential use as a patient-operated imaging tool for community-based deployment.

## 5. Conclusions

This study demonstrates the feasibility of patient-operated OCT acquisition, showing that some participants were able to obtain clinically interpretable scans using a technician-free device under supervised study conditions. While image quality metrics favored clinical Heidelberg OCT, SightSync produced sufficiently detailed retinal images to support safe referral-level decision-making, achieving strong sensitivity and a high negative predictive value. These findings highlight both the feasibility and clinical promise of patient-operated OCT as a scalable solution for expanding retinal imaging access, which, upon future iterations of the device, could be particularly useful in settings where traditional clinic-based imaging is limited. Continued refinement of hardware, real-time feedback, and image-processing algorithms will further enhance usability and diagnostic performance as the technology advances toward broader deployment.

## Data Availability

The data presented in this study are available on reasonable request from the corresponding authors. Availability is subject to the discretion of the authors.

## Supplementary Materials

The following supporting information can be downloaded at: https://www.mdpi.com/article/10.3390/bioengineering13010061/s1, Figure S1: Inclusion and exclusion criteria for study enrollment. Patients were eligible if they were receiving an eye examination in the Louis Stokes VHA Retina Clinic (Cleveland, OH). Figure S2: User-facing OCT test protocol illustrating proper device positioning and scan initiation steps. Figure S3: A Clinical setup of the patient-operated OCT device (SightSync) and B the reference clinical OCT system (Heidelberg) within the retina clinic examination rooms. Figure S4: Programmatically generated 2 × 2 collage displaying paired SightSync and clinical OCT B-scans. These collages were prepared for independent reviewer assessment of image quality and diagnostic interpretability.
