# Supplementary material for: Expanding Access to Retinal Imaging Through Patient-Operated Optical Coherence Tomography in a Veterans Affairs Retina Clinic
Source: Bioengineering (Basel). 2026 Jan 5;13(1):61. doi: 10.3390/bioengineering13010061 (PMC12837598; doi:10.3390/bioengineering13010061)
Supplement: Supplementary file 1 [file bioengineering-13-00061-s001.zip › bioengineering-4055537-supplementary.pdf]

bioengineering

MDPI

Article

Expanding Access to Retinal Imaging through Patient-Operated Optical Coherence Tomography

Supplementary Materials

Figure S1: Inclusion and exclusion criteria for study enrollment. Patients were eligible if they were receiving an eye examination in the Louis Stokes VHA Retina Clinic (Cleveland, OH).

INCLUSION CRITERIA	EXCLUSION CRITERIA
1. Patients receiving an eye exam in the retina clinic, including an OCT scan.	1. Patients with an inability to use a machine, fixate or keep head still, (i.e. hand tremors, paralysis).<br>2. Patients who are unable to assume appropriate positioning at the OCT device.<br>3. Patients with a visual acuity worse than 20/200.<br>4. Patients with significant ocular media opacities precluding an OCT scan (posterior subcapsular cataract, vitreous hemorrhage, etc.), or severe ptosis.

 https://doi.org/10.3390/bioengineering13010061

OCT TEST PROTOCOL **REV 3.0**

INSTRUCTIONS:

PLEASE READ ALL STEPS BEFORE BEGINNING TEST

1.) **USING THE HANDLE, ADJUST THE DEVICE AND PLACE EYE BEING EXAMINED ONTO THE RUBBER CUSHION. ENSURE THERE IS FULL CONTACT AROUND THE EYE.**

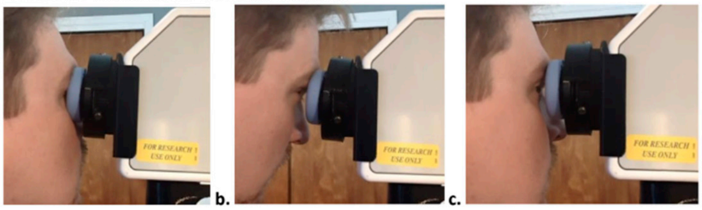

a. b. c.

GOOD **BAD** *(angled down)* **BAD** *(gap too big)*

2.) **WHEN THE SMALL RED DOT IS IN FOCUS, PRESS AND RELEASE THE RED BUTTON TO BEGIN THE TEST. THE RED DOT WILL TURN INTO A RED CROSS HAIR.**

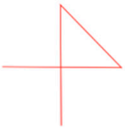

3.) **SLOWLY PRESS YOUR EYE INTO THE EYEPIECE, THEN SLOWLY RELEASE THE TENSION BACK OUT WHILE KEEPING FULL EYE CONTACT WITH THE RUBBER CUSHION. YOU SHOULD FEEL THE SPRING COMPRESS IN. TRY AND KEEP YOUR HEAD LEVEL, AND CROSSHAIR CENTERED.**

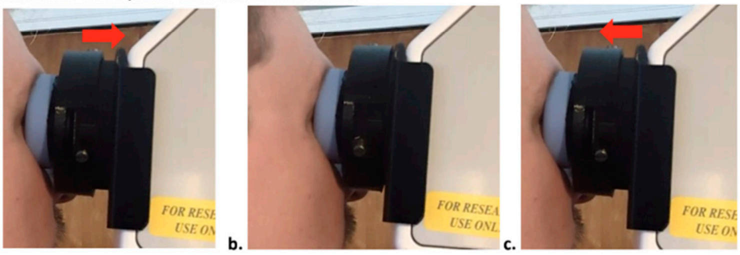

a. b. c.

4.) **AFTER A PRE-DESIGNATED TIME, THE CROSS HAIR WILL TURN BACK TO A RED DOT, INDICATING THE SCAN IS COMPLETE.**

5.) **REVIEW SCAN FOR QUALITY, AND REPEAT IF NECESSARY.**

Figure S2: User-facing OCT test protocol illustrating proper device positioning and scan initiation steps.

https://doi.org/10.3390/bioengineering13010061

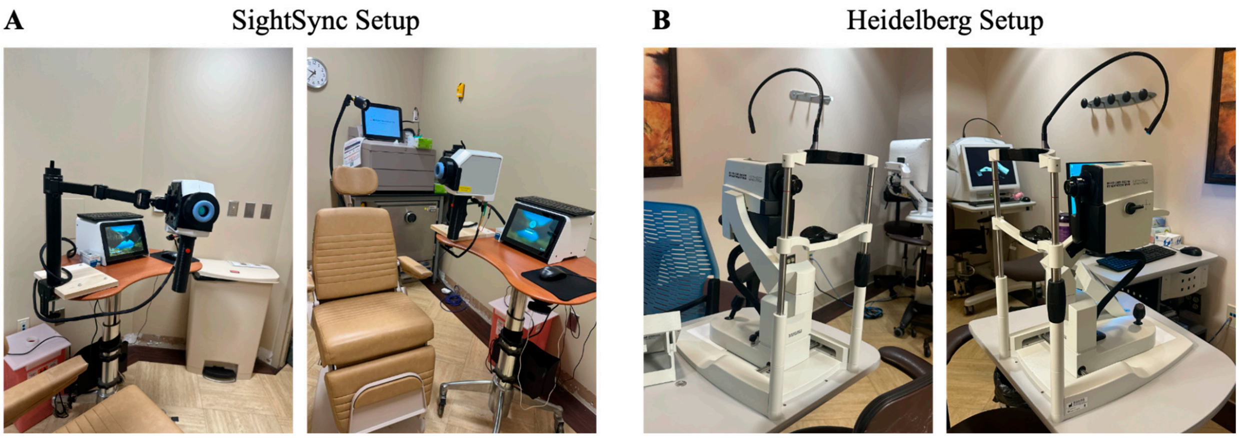

Figure S3: **A** Clinical setup of the patient-operated OCT device (SightSync) and **B** the reference clinical OCT system (Heidelberg) within the retina clinic examination rooms.

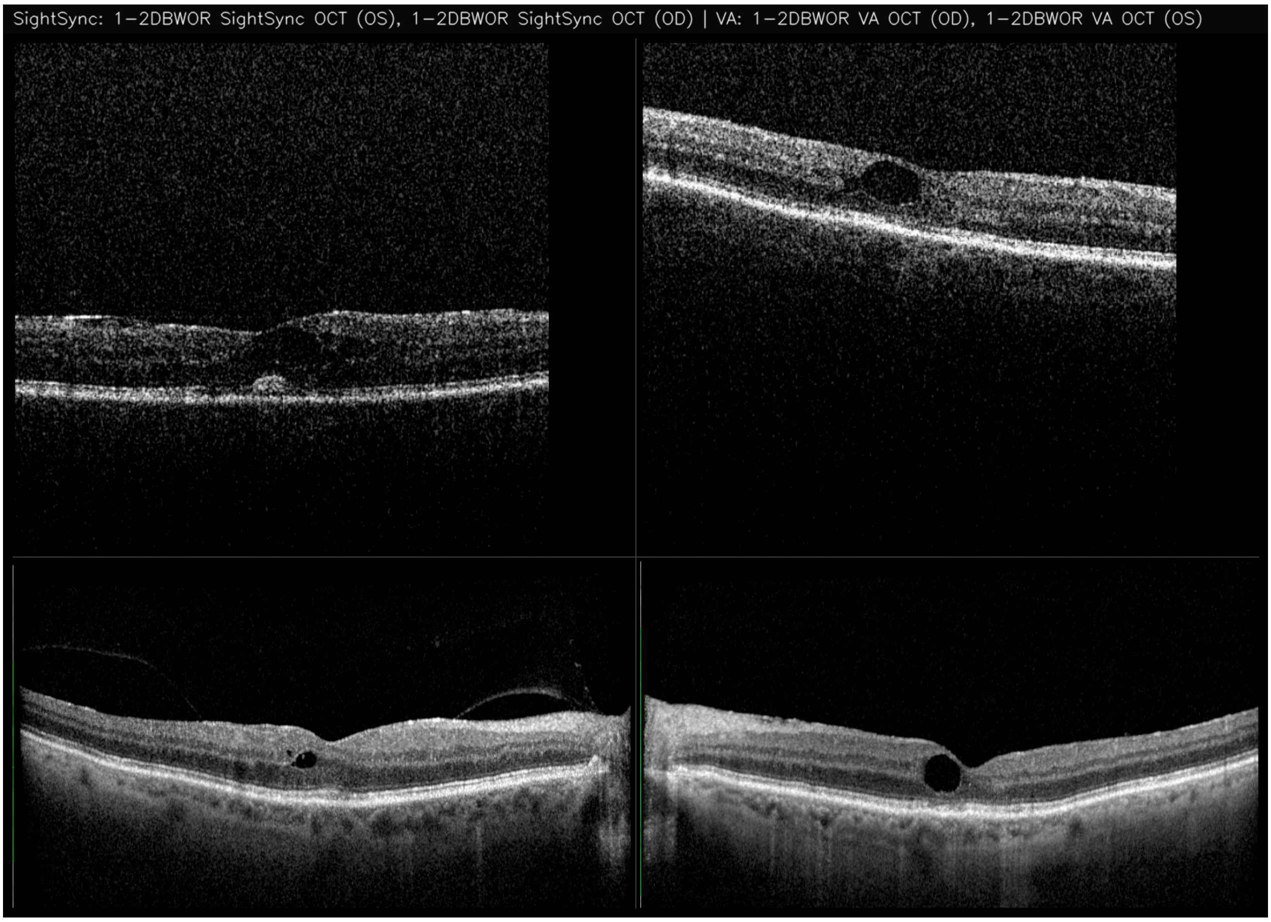

Figure S4: Programmatically generated 2×2 collage displaying paired SightSync and clinical OCT B-scans. These collages were prepared for independent reviewer assessment of image quality and diagnostic interpretability.

https://doi.org/10.3390/bioengineering13010061
